# Merkel cell carcinoma of the dorsal forearm

**DOI:** 10.1016/j.jdcr.2024.11.004

**Published:** 2024-11-25

**Authors:** Ashley Wittmer, Ronnie Youssef, Betsy Furukawa

**Affiliations:** a Texas A&M University College of Medicine, Round Rock, Texas; b Department of Dermatology, Baylor Scott & White Medical Center, Temple, Texas

**Keywords:** chrysalis, Merkel cell carcinoma, milky-red globules, polymorphous vessels

## Clinical presentation

**Fig 1:**
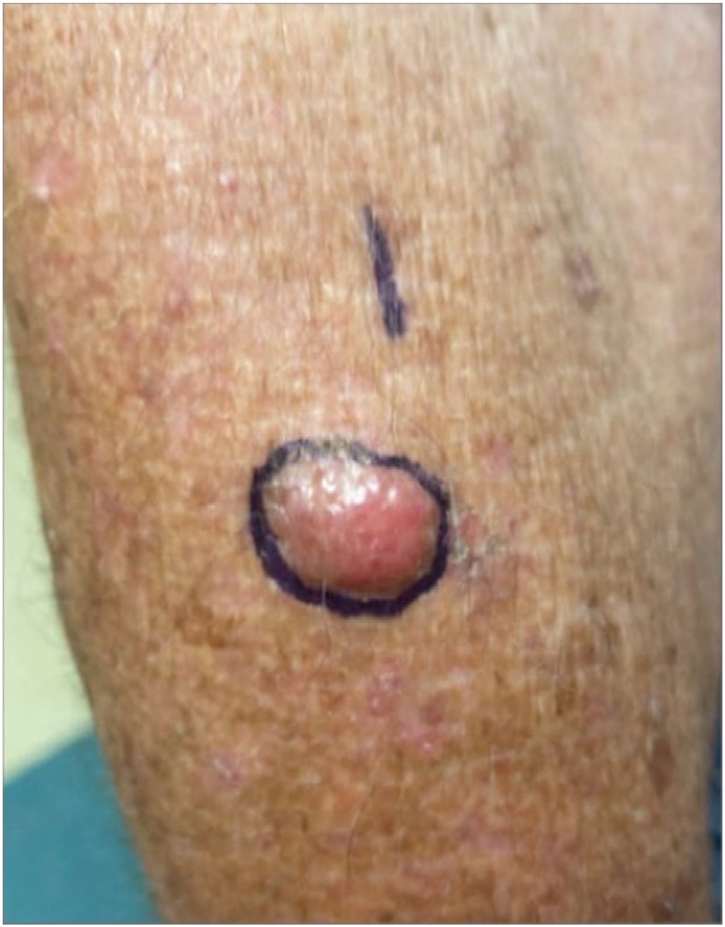
*Pink*, 1.5 cm nodule observed on the patient’s right dorsal forearm.

An 80-year-old man with a history of nonmelanoma skin cancer, presented with a 1.5 cm asymptomatic pink nodule on his right dorsal forearm that had been rapidly growing for 2-3 weeks (Fig 1).

## Dermatoscopic appearance

**Fig 2:**
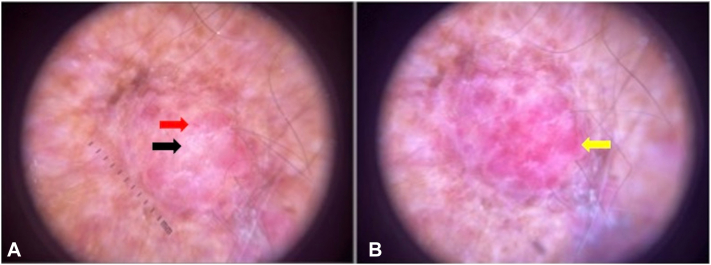
**A,** Dermoscopy revealing features of MCC including linear, *dotted*, and glomerular polymorphous vessels (*red arrow*). Shiny, *white* chrysalis structures, and scar-like depigmentation are located centrally (*black arrow*). **B,** Dermoscopic image demonstrating the *milky-red* globules (areas) that are characteristic of MCC (*yellow arrow*). *MCC*, Merkel cell carcinoma.

Dermoscopy showed linear, dotted, and glomerular polymorphous vessels in addition to milky-red globules. Scar-like depigmentation and shiny white chrysalis structures were also observed (Fig 2).

## Histologic diagnosis

Histopathologic examination from a shave biopsy revealed sheets of small, round, blue cells with high nuclear-cytoplasmic ratios, salt-and-pepper chromatin, and many mitotic figures within the dermis. CK20 staining showed a perinuclear dot-like pattern. A diagnosis of cutaneous high-grade neuroendocrine Merkel cell carcinoma (MCC) was rendered. A PET/CT scan was normal, but sentinel node biopsy revealed one positive axillary node. He underwent wide local excision followed by external beam radiation therapy to the right forearm and right axilla to a dose of 5600 cGy in 28 fractions. Subsequently, he was treated with 3 cycles of pembrolizumab 200 mg IV infusions.

**Key message**

MCC is an aggressive, rare neuroendocrine carcinoma. It commonly presents as a solitary, rapidly expanding nodule or plaque that is typically erythematous or violaceous with a shiny, smooth appearance. These lesions often occur in sun-exposed areas, most frequently effecting the head, neck, and limbs. The risk for developing MCC increases with age and is higher for males compared to females. Pertinent risk factors for developing MCC include having a lighter skin type, history of heavy UVB exposure, and immunosuppression.^1^ Common dermoscopy findings of MCC include polymorphous vessels and milky-red or pink-white structureless areas.^2^ The presence of polymorphous vessels and milky-red areas may lead to the inclusion of amelanotic melanoma in the differential diagnoses.^2^ Dermoscopy plays a vital role in the early detection and differentiation of MCC from other tumors. Notably, our case demonstrates shiny white chrysalis structures, which are a rare dermoscopic finding in MCC whose significance is not well understood.^2^ Our case highlights the importance of these structures in distinguishing MCC from other dermatological conditions.

## Conflicts of interest

None disclosed.
